# Meta-analysis of the association of HLA-DRB1 with rheumatoid arthritis in Chinese populations

**DOI:** 10.1186/1471-2474-14-307

**Published:** 2013-10-27

**Authors:** Meng Yang, Xiaocong Kuang, Jianmin Li, Yanbin Pan, Meile Tan, Binzhu Lu, Qiumei Cheng, Lingyan Wu, Guodong Pang

**Affiliations:** 1 Postgraduate School of Guangxi Medical University, 22 Shuangyong Road, Nanning 530021, Guangxi, People’s Republic of China; 2 Department of pathophysiology of Guangxi Medical University, 22 Shuangyong Road, Nanning 530021, Guangxi, People’s Republic of China; 3 Department of Dermatology, the third affiliated hospital of Guangxi Medical University, 13 Dancun Road, Nanning 530031, Guangxi, People’s Republic of China

**Keywords:** Rheumatoid arthritis, HLA-DRB1, Laboratory parameters, Meta-analysis

## Abstract

**Background:**

Individual studies have reported different results regarding the association of HLA alleles with RA in Chinese populations. This study was performed to systematically summarize results on the association of HLA-DRB1 with rheumatoid arthritis (RA) in China.

**Methods:**

We examined the case–control studies concerned about the relationship between HLA-DRB1and RA and differences of clinical and laboratory parameters between the HLA-DR4 (DR4)+ and DR4- in RA patients in Chinese populations. Odds ratios (ORs) and weighted mean difference (WMD) with corresponding 95% confidence intervals (CI) was used to describe the relationship.

**Results:**

22 studies with 1690 cases and 1793 controls were included. Chinese populations with RA had significantly higher frequencies of HLA-DRB1*04, *0401, *0404, *0405 and *0410 than controls (ORDRB1*04 =4.19, 95% CI =3.44–5.11, p<0.00001; ORDRB1*0401 =2.53, 95% CI =1.54–4.16, p=0.0003; ORDRB1*0404 =2.28, 95% CI =1.28–4.06, p=0.005; ORDRB1*0405=3.71, 95% CI =2.52–5.45, p<0.00001; ORDRB1*0410 =2.99, 95% CI =1.25–7.14, p=0.01respectively). As to laboratory parameters, Erythrocyte sedimentation rate (ESR), C-reactive protein (CRP), Rheumatoid factor (RF), Anti-cyclic citrullinated peptide antibodies (Anti-CCP ) in patients with DR4+ were higher than patients with DR4- (WMD=0.26, 95% CI =0.15–0.37, p<0.00001; WMD = 0.26, 95% CI =0.12–0.41, p=0.0005; WMD = 0.44, 95% CI =0.23–0.65, p<0.00001; WMD = 0.58, 95% CI =0.24–0.91, p=0.0007 respectively). As to clinical features, there was no difference in duration of morning stiffness, number of swollen joints, number of joint tenderness, X-ray phases and joint function between the DR4+ and DR4- in RA patients.

**Conclusions:**

It was found that HLA-DRB1*04, *0401, *0404, *0405 and *0410 are risk factors for RA in Chinese populations. ESR, CRP, RF, Anti-CCP are different between the DR4+ and DR4- in RA patients in Chinese populations, while there’s no difference for indexes of clinical features.

## Background

RA is an autoimmune disease characterized by chronic inflammation of the joints, which may lead to joint destruction and disability. It is the common chronic inflammatory rheumatic disease in the world, with prevalence estimates of 0.25% to 0.5% [1]. Previous literature suggests that people with rheumatoid arthritis may live 10–15 years less than their healthy counterparts [2]. RA is a complex polygenic disease of unknown etiology. However, genetic variation is believed to be important in determining the susceptibility of RA. It was proven to be associated with (histocompatibility locus antigen) HLA region strongly, especially with HLA-DRB1 alleles [3].

HLA-DRB1 alleles encode (70Q(R)K(R)RAA74) encoding the shared epitope (SE) (RAA amino acid pattern in positions 72 to 74 of the third hypervariable region of the DRβ1 chain) are associated with RA susceptibility [4]. SE contains HLA-DRB1 alleles representing significant genetic risk factor for RA. Many studies have attempted to clarify the relationship between HLA-DRB1 and RA, but there has been no definite consensus to date in Chinese populations. Due to the relatively small numbers of patients or the ethnic and clinical heterogeneity of the patients, the results are different in many studies. Well-designed meta-analyses of Caucasian and American populations showed that there was a strong association between HLA-DRB1 and RA susceptibility and severity [5].

Individual studies have reported different results regarding the association of HLA alleles with RA in Chinese populations. Some have reported that the frequency of the HLA-DRB1*0401 and *0405 alleles are significantly increased in RA patients, whereas others have found no associations [6,7]. Moreover, there were controversial results about differences of clinical and laboratory parameters between the DR4+ and DR4- in RA patients [8,9].

This study was performed to systematically summarize the association between Chinese with RA and HLA-DRB1 alleles. It was also performed to investigate the differences of clinical and laboratory parameters between the DR4+ and DR4- in RA patients. The frequency of HLA-DRB1 alleles varies according to ethnic and racial background, with some alleles being extremely rare. Therefore, articles were not required to identify all alleles for inclusion.

## Methods

### Literature search and selection

The following databases: Cochrane Library, PubMed, Embase, Chinese BioMedical Literature Database (CBM), China National Knowledge Infrastructure (CNKI), WANFANG and Chinese Social Sciences Citation Index(VIP) databases were searched for available articles without language restrictions. The index terms that we used were: (“human leukocyte antigen” or “HLA”) and (“rheumatoid arthritis” or “RA”) and (“Chinese” or “China”). Last query was updated on 31 March, 2013. References of retrieved articles were cross-searched to identify any studies missed by the electronic search strategies.

### Inclusion criteria

The studies which were fit into the meta-analysis was required to meet the following criteria: (1) the articles must be proven diagnosis of RA patients with standard criteria of American College of Rheumatology (ACR) in 1987. (2) Study should be correlated with the RA and HLA-DRB1 in Chinese populations. (3) Study must be case–control design. (4) When multiple articles were published by the same authors or institutions, the most recent or informative single article was selected. (5) Articles lacking original data for meta-analysis, review articles were excluded.

### Quality assessment of included studies

The quality of papers was independently assessed by two reviewers (Pan Yanbin and Tan Meile) based on the STROBE quality score systems [10]. Quality scores ranged from 0 to 30. We defined 10, 20 and 30 scores as low, moderate and high grade respectively. Articles with quality scores of 10 or less were excluded. Any discrepancies between the two reviewers were resolved by discussion and consultation with a third reviewer.

### Data extraction

Data for this meta-analysis were extracted by two investigators (Li Jianmin and Wu Lingyan) independently and reached a consensus on the disagreement. Results would be arbitrated by a third reviewer (Yang Meng) if there were any disagreement. The extracted details included first author, year of publication, geographical region,study design, sample, source of cases and controls, frequency of HLA-DRB1 alleles, number of cases and controls, clinical feature, laboratory index and detection methods. The X-ray phases was according to standard of Steinbrocker classification for the joint function and joint function was measured by justification of triaxial goniometer. These geographical populations can be classified into northern Han Chinese (N-Han) and southern Han Chinese (S-Han), with the Yangtze River used as a geographical boundary. In the subgroup analysis, subjects of all included studies were divided into the southerners and northerners of China due to significantly geographic variation in Chinese populations.

### Statistical analysis

Meta-analysis was performed using the Review Manager version 5.2 (provided by The Cochrane Collaboration) and STATA package version 12.0 (Stata Corporation, College Station, TX, USA). Dichotomous data were reported as OR (calculated by chi-square test) whereas continuous data were reported as WMD ± standard deviation (SD) (calculated by T test). The pooled OR or WMD together with the 95% confidence interval (CIs) were used for assessing the strength of association. The heterogeneity among studies was judged by I [2] statistics. It ranges between 0 and 100% and I [2] values of 25, 50 and 75% were defined as low, moderate and high estimates, respectively. When a significant I [2] >50% indicated heterogeneity across studies, the random effects model was used for meta-analysis, or else the fixed effects model was used [11]. Besides, sensitivity analyses were utilized to evaluate the stability of the results. And the potential publication bias was assessed by the Begg's funnel plot and Egger's test [12]. Statistical significance was considered when the P value of the test was <0.05. All P values were two-sided.

## Results

**Figure 1:**
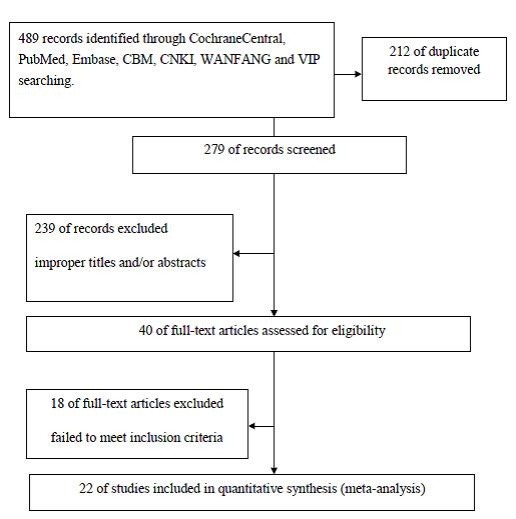
**Selection process for the studies included in the Meta**-**analysis.**

With our inclusion criteria, a total of 489 references were retrieved for initial review using search strategies as described. 239 citations were excluded from analysis after the first screening based on abstracts or titles. After exclusion of the articles that did not meet the inclusion criteria, we identified 40 potential studies for detail evaluation. Upon further review, 18 articles were eliminated due to the following reasons: 3 studies overlapped with others; 15 studies didn’t provide sufficient data about HLA-DRB1 alleles frequency on cases and/or controls or lacked clinical feature or laboratory indexes data to create for meta-analysis. Finally, 22 studies performed on the association of HLA-DRB1 with rheumatoid arthritis in Chinese populations were included and analyzed. Selection process for the studies included in the meta-analysis is summarized in Figure 1. The main features of eligible studies in our meta-analysis are summarized in Tables 1.

**Table 1:** **Main characteristics of the 22 studies included in the final meta**-**analysis**

**Author**(**reference**)	**Year**	**Region**	**Source of cases**	**Source of controls**	**Detection methods**	**Number of cases**	**Number of controls**	**Quality score**
Ye SX [13]	1989	Liaoning	Hospital-based	Population-based	NS	39	100	22
Molkentin, J [14]	1993	Shanghai	Hospital-based	Population-based	PCR-SSP	23	21	26
Zhu NS [15]	1994	Shanghai	Hospital-based	Population-based	PCR-RFLP	95	130	24
Wang BL [16]	1996	Anhui	Hospital-based	Population-based	PCR-SSP	30	30	17
Xiao Z [17]	1997	Inner Mongolia	Hospital-based	Population-based	NS	41	50	16
Zhao Y [18]	1997	Beijing	Hospital-based	Population-based	PCR-SSP	86	106	21
Gu JR [19]	1998	Guangdong	Hospital-based	Population-based	PCR-SSP	47	108	23
Yuan GH [8]	1998	Beijing	Hospital-based	Population-based	PCR-RFLP	35	100	23
Zhang JQ [20]	1999	Hebei	Hospital-based	Population-based	PCR-SSP	180	100	25
Liu QH [6]	2000	Hubei	Hospital-based	Population-based	PCR-SSP	78	126	26
Wu H [21]	2001	Hunan	Hospital-based	Population-based	PCR-SSP	105	76	32
Lin L[22]	2001	Guangdong	Hospital-based	Population-based	PCR-SSP	117	100	21
Liu YJ [23]	2002	Guizhou	Hospital-based	Population-based	PCR-SSP	35	51	22
Li XF [9]	2003	Shandong	Hospital-based	Population-based	PCR-SSP	132	130	31
Zhang SL[24]	2003	Fujian	Hospital-based	Population-based	PCR-SSP	98	100	21
Su Y [25]	2004	Beijing	Hospital-based	Hospital-based	PCR-SSP	136	79	24
Duan JM [26]	2005	Anhui	Hospital-based	Population-based	PCR-SSP	25	77	23
Zhang HW [7]	2005	Guangdong	Hospital-based	Population-based	PCR-SSP	22	31	22
Chen CP [27]	2010	Shanxi	Hospital-based	Population-based	PCR-SSP	42	14	21
Wang CF [28]	2010	Gansu	Hospital-based	Population-based	PCR-SSP	27	46	21
Chang ZF [29]	2012	Inner Mongolia	Hospital-based	Population-based	PCR-SSP	140	100	23
Shi YM [30]	2012	Xinjiang	Hospital-based	Population-based	PCR-SSP	157	118	21

'*PCR*-*SSP*’ stands for Polymerase Chain Reaction-Sequence-Specific Primers, '*PCR*-*RFLP*’ stands for Polymerase Chain Reaction-Restriction Fragment Length Polymor Phism and '*NS*’ corresponds to not stated.

### Association of DRB1 alleles with RA susceptibility

These 22 case–control studies selected included a total of 1690 RA cases and 1793 healthy controls. A total of 12 alleles were assayed in the studies: HLA-DRB1*01,*0101,*0102(DR1); HLA-DRB1*04,*0401,*0402,*0404,*0405,*0408,*0409 and *0410(DR4); HLA-DRB1*10(DR10). The most common polymorphisms were DRB1*0405. Summary of the meta-analysis findings of the association between HLA-DRB1 alleles and RA is shown in Table 2.

**Table 2:** **Summary of the meta**-**analysis findings of the association between HLA**-**DRB1 alleles and RA**

**Polymorphisms**	**Eligible studies**	**RA Case n**/**N**	**Control n**/**N**	**OR (95%** **CI)**	**p**–**value**	**Heterogeneity test**	**Effect model**
HLA–DRB1*04	12	489/965	240/1166	4.19 (3.44 – 5.11)	<0.00001	p=0.93, I [2]=0%	Fixed
Northerner	5	248/528	111/566	3.67 (2.78 – 4.84)	<0.00001	p=0.96, I [2]=0%	
Southerner	7	241/437	129/600	4.83 (3.64 – 6.4)	<0.00001	p=0.86, I [2]=0%	
HLA–DRB1*0401	10	54/733	24/814	2.53 (1.54 – 4.16)	0.0003	p=0.91, I [2]=0%	Fixed
Northerner	5	31/397	16/390	2 (1.06 – 3.79)	0.003	p=0.87, I [2]=0%	
Southerner	5	23/336	8/424	3.59 (1.6 – 8.06)	0.002	p=0.78, I [2]=0%	
HLA–DRB1*0402	1	4/287	2/196	1.41 (0.31 – 6.42)	0.65	p=0.75, I [2]=0%	Fixed
HLA–DRB1*0404	10	44/733	19/814	2.28 (1.28 – 4.06)	0.005	p=0.91, I [2]=0%	Fixed
Northerner	5	31/397	10/390	2.46 (1.14 – 5.28)	0.02	p=0.75, I [2]=0%	
Southerner	5	13/336	9/424	2.06 (0.85 – 4.94)	0.11	p=0.72, I [2]=0%	
HLA–DRB1*0405	11	240/913	91/914	3.71 (2.52 – 5.45)	<0.00001	p=0.10, I [2]=38%	Random
Northerner	6	142/577	54/490	3.17 (1.84 – 5.48)	<0.00001	p=0.11, I [2]=44%	
Southerner	5	98/336	37/424	4.56 (2.69 – 7.73)	<0.00001	p=0.26, I [2]=25%	
HLA–DRB1*0408	9	16/620	7/757	2.24 (0.94 – 5.34)	0.07	p=0.77, I [2]=0%	Fixed
Northerner	5	13/295	5/350	2.27 (0.82 – 6.29)	0.12	p=0.51, I [2]=0%	
Southerner	4	3/325	2/407	2.18 (0.42 – 11.3)	0.35	p=0.63, I [2]=0%	
HLA–DRB1*0409	8	8/578	3/743	2.78 (0.92 – 8.42)	0.07	p=0.43, I [2]=0%	Fixed
Northerner	4	6/253	2/336	3.28 (0.85 – 12.57)	0.08	p=0.22, I [2]=35%	
Southerner	4	2/325	1/407	1.91 (0.26 – 13.95)	0.53	p=0.93, I [2]=0%	
HLA–DRB1*0410	8	15/578	7/743	2.99 (1.25 – 7.14)	0.01	p=0.93, I [2]=0%	Fixed
Northerner	4	4/253	1/336	3.96 (0.71 – 22.12)	0.12	p=0.92, I [2]=0%	
Southerner	4	11/325	6/407	2.7 (0.98 – 7.42)	0.05	p=0.58, I [2]=0%	
HLA–DRB1*01	8	45/557	45/785	1.35 (0.87 – 2.11)	0.18	p=0.87, I [2]=0%	Fixed
Northerner	4	25/292	27/436	1.45 (0.8 – 2.61)	0.22	p=0.63, I [2]=0%	
Southerner	4	20/265	18/349	1.23 (0.62 – 2.45)	0.55	p=0.72, I [2]=0%	
HLA–DRB1*0101	1	9/245	9/356	1.48 (0.57 – 3.84)	0.42	p=0.92, I [2]=0%	Fixed
HLA–DRB1*0102	1	4/245	5/356	1.08 (0.3 – 3.9)	0.9	p=0.95, I [2]=0%	Fixed
HLA–DRB1*10	5	20/417	18/538	1.3 (0.67 – 2.52)	0.43	p=0.69, I [2]=0%	Fixed
Northerner	3	16/253	14/336	1.34 (0.64 – 2.84)	0.44	p=0.44, I [2]=0%	
Southerner	2	4/164	4/202	1.17 (0.29 – 4.71)	0.82	p=0.43, I [2]=0%	

'*n*’ stands for number of positive events, '*N*’ stands for number of total events.

**Figure 2:**
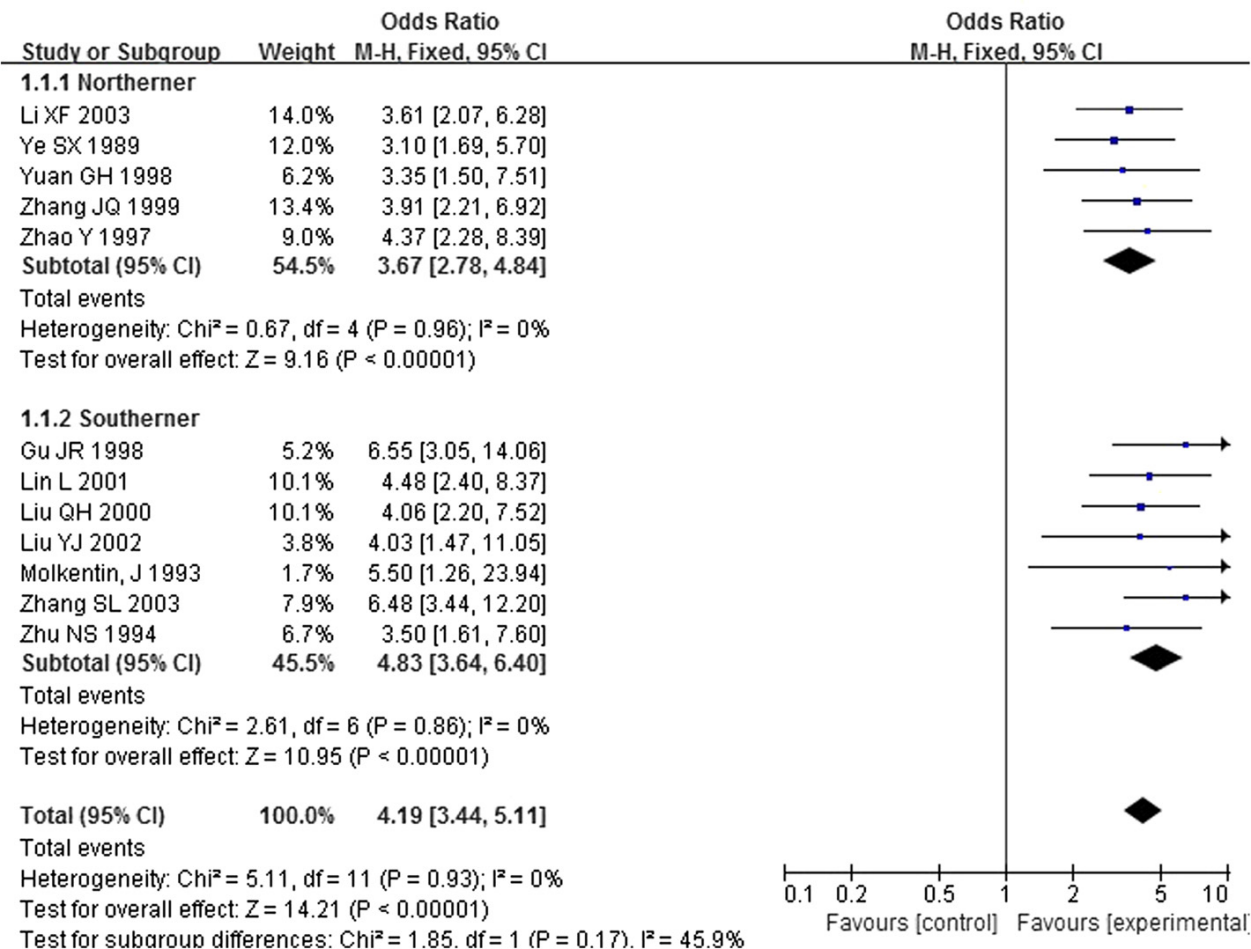
**Meta analysis of the association of HLA**-DRB1*04 with rheumatoid arthritis in Chinese populations.

**Figure 3:**
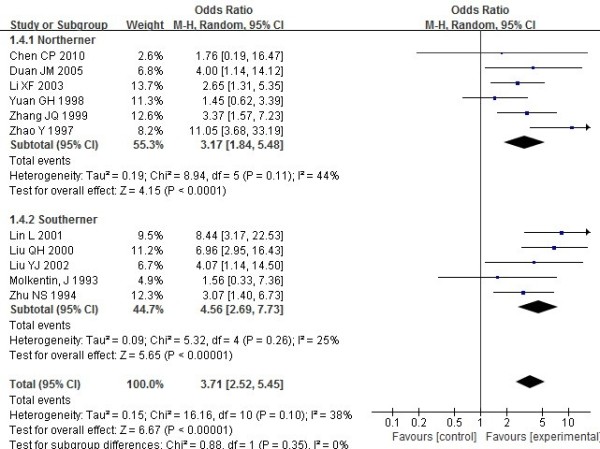
**Meta analysis of the association of HLA**-DRB1*0405 with rheumatoid arthritis in Chinese populations.

The ORs showed that Chinese patients with RA had significantly higher frequencies of HLA-DRB1*04 (Figure 2), *0401, *0404, *0405 (Figure 3) and *0410 (OR_DRB1*04_ =4.19, 95% CI =3.44–5.11, p<0.00001; OR_DRB1*0401_ =2.53, 95% CI =1.54–4.16, p=0.0003; OR_DRB1*0404_=2.28, 95% CI =1.28–4.06, p=0.005; OR_DRB1*0405_=3.71,95%CI=2.52–5.45, p<0.00001; OR_DRB1*0410_=2.99,95% CI=1.25–7.14, p=0.01 respectively).

However, HLA-DRB1*0408,*0409,*01,*0101,*0102 and *10 showed no association with susceptibility to RA in Chinese populations (OR_DRB1*0408_ =2.24, 95% CI =0.94–5.34, p=0.07; OR_DRB1*0409_ =2.24, 95% CI =0.94–5.34, p=0.07; OR_DRB1*01_ =1.35, 95% CI =0.87–2.11, p=0.18; OR_DRB1*0101_ =1.48, 95% CI=0.57–3.84, p=0.42; OR_DRB1*0102_=1.08,95%CI=0.3–3.9, p=0.9; OR_DRB1*10_=1.3, 95% CI =0.67–2.52, p=0.43 respectively).

Results of subgroup analysis showed that there was a positive association between DRB1*0404 and susceptibility to RA in the northerners (OR=2.46, 95% CI=1.14–5.28), but not in the southerners (OR =2.06, 95% CI=0.85–4.94). Results of subgroup analysis showed that there was a higher susceptibility of DRB1*04,*0401,*0405 in the southerners than in the northerners of China. No association was found between DRB1*0408, *0409, *01,*0101, *0102 and *10 and susceptibility to RA both in the southerners and northerners of China.

### Differences of clinical and laboratory parameters between the DR4+ and DR4- in RA patients

**Figure 4:**
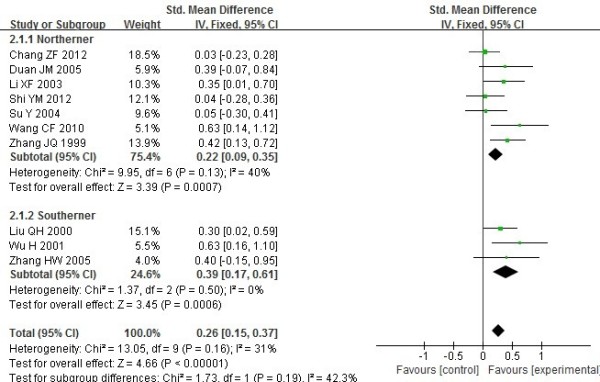
**Meta analysis of the differences of ESR between the DR4**+ **and DR4**- **in RA patients.**

**Figure 5:**
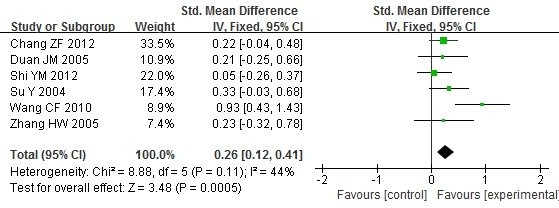
**Meta analysis of the differences of CRP between the DR4**+ **and DR4**- **in RA patients.**

Chi-square test or T test was took to evaluate the balance of average age, gender and disease duration of included studies, there were not obvious imbalance of the baseline for them. We contacted the original authors to get data of treatments and sensitivity of the scale of X-ray. There were not obvious imbalance of the baseline for them too. A summary of the meta-analysis findings of the association between DR4+ and DR4-in RA patients is shown in Table 3. As to laboratory parameters, ESR (Figure 4), CRP (Figure 5), RF, Anti-CCP in patients with DR4+ were higher than patients with DR4- (WMD=0.26, 95% CI =0.15–0.37, p<0.00001; WMD = 0.26, 95% CI =0.12–0.41, p=0.0005; WMD = 0.44, 95% CI =0.23–0.65, p<0.00001; WMD = 0.58, 95% CI =0.24–0.91, p=0.0007 respectively).

**Table 3:** **Summary of the meta**-**analysis findings of the differences of clinical and laboratory parameters between the DR4**+ **and DR4**- **in RA patients**

**Polymorphisms**	**Eligible studies**	**Baseline comparability**	**Case (N or n**/**N) DR4**+	**Control** (**N or n**/**N**) **DR4**-	**WMD**/**OR (95%** **CI)**	**p**–**value**	**Heterogeneity test**	**Effect model**
ESR	10	Yes	590	762	0.26 (0.15, 0.37)	<0.00001	p=0.16, I [2]=31%	Fixed
Northerner	7	Yes	458	562	0.22 [0.09, 0.35]	0.0007	p=0.13, I [2]=40%	
Southerner	3	Yes	132	200	0.39 [0.17, 0.61]	0.0006	p=0.16, I [2]=31%	
CRP	6	Yes	325	436	0.26 [0.12, 0.41]	0.0005	p=0.11, I [2]=44%	Fixed
RF	4	Yes	139	246	0.44 [0.23, 0.65]	<0.00001	p=0.42, I [2]=0%	Fixed
Anti-CCP	2	Yes	52	123	0.58 [0.24, 0.91]	0.0007	p=0.28, I [2]=15%	Fixed
Duration of morning stiffness	5	Yes	228	337	0.23 [-0.01, 0.48]	0.06	p=0.11, I [2]=47%	Fixed
Number of swollen joints	7	Yes	414	527	0.13 [-0.00, 0.26]	0.05	p=0.29, I [2]=18%	Fixed
Number of joint tenderness	6	Yes	368	437	0.08 [-0.06, 0.23]	0.25	p=0.67, I [2]=0%	Fixed
X-ray phases	8	Yes	137/368*	142/365*	0.93 (0.66–1.29)’	0.65	p=0.1, I [2]=42%	Fixed
I ~ II	3	Yes	44/108*	63/135*	0.47 (0.26–0.87)’	0.02	p=0.75, I [2]=0%	
III ~ IV	5	Yes	93/260*	79/230*	1.27 (0.84–1.92)’	0.25	p=0.26, I [2]=25%	
Joint function	8	Yes	93/260*	79/230*	0.89 (0.65–1.23)’	0.6	p=0.02, I [2]=58%	Random
I ~ II	3	Yes	124/364*	182/463*	0.59 (0.35–1)’	0.05	p=0.51, I [2]=0%	
III ~ IV	5	Yes	56/149*	96/170*	1.14 (0.76–1.69)’	0.88	p=0.02, I [2]=65%	

'*n*’ stands for number of positive events, '*N*’ stands for number of total events.

* n/N.

'OR (95%CI).

**Figure 6:**
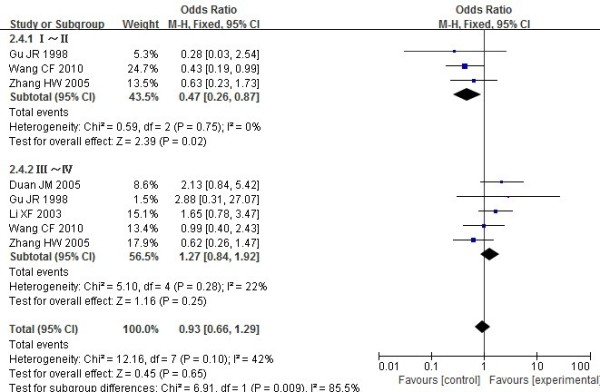
**Meta analysis of the differences of X**-**ray phases between the DR4**+ **and DR4**- **in RA patients.**

As to clinical features, there was no difference in duration of morning stiffness, number of swollen joints and number of joint tenderness between the DR4+ and DR4- in RA patients (WMD=0.23,95% CI=-0.01–0.48, p=0.06;WMD=0.13,95% CI=-0.00–0.26, p=0.05;WMD=0.08,95% CI=-0.06–0.23, p=0.25 respectively). There was no difference in X-ray phases (Figure 6) and joint function between the DR4+ and DR4- in RA patients (OR =0.93, 95% CI =0.66–1.29, p=0.65; OR = 0.89, 95% CI =0.65–1.23, p=0.6 respectively).

Results of subgroup analysis showed that there was a positive association between DRB1*0404 and X-ray phases to RA in phases I~II (OR=0.47, 95% CI=0.26–0.87), but not in the phases III~IV (OR =1.27, 95% CI=0.84, 1.92).

### Sensitivity analysis and publication bias

**Figure 7:**
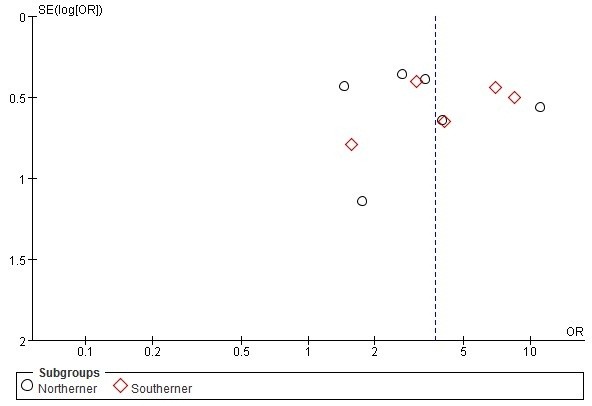
**Funnel plot for the association of HLA**-DRB1*0405 with rheumatoid arthritis in Chinese populations.

The sensitivity analyses revealed that none of the studies significantly affected the pooled ORs and CIs.When each study is sequentially removed and the pooled ORs remain almost the same. The funnel plot to detect publication bias showed relatively symmetric for most alleles (e.g. Figure 7 Funnel plot for the association of HLA-DRB1*0405 with rheumatoid arthritis in Chinese populations), except for ESR. Moreover, the regression asymmetry test did not reveal significant evidence for publication bias (Egger p=0.348).

## Discussion

In this meta-analysis, 12 HLA-DRB1 polymorphisms were addressed and evaluated. Our meta-analysis results showed that there was a positive association between HLA-DRB1*04, *0401, *0404, *0405 and *0410 and susceptibility to RA, which indicated that they may be potential risk factors for RA in Chinese populations. The SE hypothesis indicated that these genes encoding the third hypervariable region of the DR antigen amino acid and the sequence are QKRAA, or QRRAA or RRRAA [31]. It is based on the assumption that class II molecules are directly involved in the etiology and pathogenesis of autoimmune disease. The initiation of an immune response requires T cell activation, and such activation requires the presence of both antigen and HLA-DRB1 on antigen presenting cells. Many current studies assume that some direct physical interaction between class II molecules and T cell receptor or antigen is involved in this process. The SE hypothesis assumes that structural differences between class II molecules can influence the interaction, either at the level of antigen presentation or during T cell differentiation in the thymus [32].

Teller found that the “shared” DRB1 amino acid sequence was relatively infrequent in the Hispanic American patients with RA which may not be useful in other ethnic groups [33]. This study may supplement data of association of DRB1 in Chinese populations.

In the subgroup analysis by geographic distribution, results showed that DRB1*0404 might be a potential risk factor for RA in the northerners of China rather than southerners. It showed that there was a higher susceptibility of DRB1*04,*0401,*0405 in the southerners than in the northerners of China. However as to DRB1*0404, it had an opposite result. All these may suggest a possible role of geographic differences in genetic backgrounds and the environment they lived in.

Summary of the meta-analysis findings of the association between DR4+ and DR4-in RA patients was shown in Table 3. As to laboratory parameters, ESR, CRP, RF, Anti-CCP in patients with DR4+ were higher which indicate the serious condition, poor prognosis and difficulty to alleviate in patients with DR4+. As to clinical features, there was no difference in duration of morning stiffness, number of swollen joints, joint function and number of joint tenderness between the DR4+ and DR4- in RA patients, the mechanism about it should be further explored.

Results of subgroup analysis show that there was a positive association between DRB1*0404 and X-ray phases to RA in phases I~II, but not in the phases III~IV. It suggests that the DR4+ patients with bone erosion may occur at an early stage.

We also found that the most common polymorphism was DRB1*0405 in Chinese population. While DRB1*0401 and DRB1*0402 were the most common polymorphismss in white population and Jews [34]. The role of the various subtypes in RA susceptibility were different in different populations and the constitute of susceptibility and non-susceptibility subsets were also different. Therefore, studies of RA-related genes should be on the basis of region, nationality and the sample should be expanded and more associated genes should be tested at the same time. We should also improve the typing technology, making a comprehensive analysis of the patients with RA. This will benefit to the revealment of the pathogenesis, early diagnosis and the prevention and control of RA.

Several limitations of the current studies could not be ignored. First, although we did not detect significant publication bias except for ESR between studies, it is uncertain whether the cases are comparably representative, and they are observational studies, more prone to many biases than prospective randomized controlled studies [35]. We found publication bias for ESR, there may be missing information which reflected “negative” or more conservative association of ESR with DR4.It need more samples to validate the reliability of our conclusions. Secondly, we distinguish the geographical populations into N-Han and S-Han with the Yangtze River serving as a geographical boundary, however the way to distinguish them is complicated which may lead to an inflated rate of false-positive results [36]. Thirdly, we were not able to perform subgroup for each polymorphism due to the limited number of published studies. Fourthly, our systematic review was based on unadjusted data, as the alleles information stratified for the main confounding variables was not available in the original papers. Although we actively contacted with the authors, we did not got a comprehensive set of data. Fifthly, we were not able to stratify our analyses according to the presence of anti-CCP antibodies. Chun-Lai found that different DRB1 SE alleles are common in Asian patients. These alleles confer a significant risk of developing ACPA-positive RA, but not ACPA-negative RA (i.e., in three Asian populations from Malaysia) [37]. Fisher et al. found that HLA-DRB1 SE was associated with both ACPA positive and negative RA, in a Korean population, but that there was a particularly strong interaction of smoking and HLA- DRB1 SE alleles that associated with ACPA fine-specificity [38,39]. Finally, although all cases and controls of each study were well defined with similar inclusion criteria, there may be potential factors that were not taken into account that may have influenced our results.

As not enough studies were available in this field and current evidence remains limited. Therefore, it should be emphasised the necessity to conduct more studies with an adequate methodological quality, properly controlling for possible confounds in order to obtain a more valid result.

## Conclusions

Our meta-analysis shows that HLA-DRB1*04, *0401, *0404, *0405 and *0410 are associated with RA, while DRB1*0405 is the most common polymorphism in Chinese populations. ESR, CRP, RF, Anti-CCP in patients with DR4+ were higher than DR4-, whereas there’s no difference for indexes of clinical features.

## Competing interests

The authors declare that they have no competing interests.

## Authors’ contributions

YM and KX carried out the literature search and wrote the manuscript. LJ and WL reviewed titles, abstracts and papers for inclusion in the review. PY and TM assessed the quality of paper based on the STROBE quality score systems. LB and CQ did the statistical analysis. PG participated in its design and helped to draft the manuscript. All authors read and approved the final manuscript.

## Pre-publication history

The pre-publication history for this paper can be accessed here:

http://www.biomedcentral.com/1471-2474/14/307/prepub
